# A new testing platform using fingerstick blood for quantitative antibody response evaluation after SARS-CoV-2 vaccination

**DOI:** 10.1080/22221751.2021.2023328

**Published:** 2022-01-07

**Authors:** Jinwei Du, Dayu Zhang, Joseph A. Pathakamuri, Daniel Kuebler, Ying Yang, Yulia Loginova, Eric Chu, Roberta Madej, Jocelyn V. Neves, Brianna Singer, Holly Radke, Naomi Spencer, Elizabeth Rizk, Aiguo Zhang, Chuanyi M. Lu, Michael Y. Sha

**Affiliations:** a DiaCarta Inc., Pleasanton, CA, USA; b Department of Biology, Franciscan University of Steubenville, Steubenville, OH, USA; c Department of Laboratory Medicine, University of California and VA Health Care System, San Francisco, CA, USA

**Keywords:** COVID19, SARS-CoV-2, anti-SARS-CoV-2 igG, fingerstick blood, quantitative immunoassay. SARS-CoV-2 vaccination

## Abstract

Testing and vaccination have been major components of the strategy for combating the ongoing COVID-19 pandemic. In this study, we have developed a quantitative anti-SARS-CoV-2 spike (S1) IgG antibody assay using a fingerstick dried blood sample. We evaluated the feasibility of using this high-throughput and quantitative anti-SARS-CoV-2 spike (S1) IgG antibody testing assay in vaccinated individuals. Fingerstick blood samples were collected and analyzed from 137 volunteers before and after receiving the Moderna or Pfizer mRNA vaccine. Anti-SARS-CoV-2 S1 IgG antibody could not be detected within the first 7 days after receiving the first vaccine dose, however, the assay reliably detected antibodies from day 14 onwards. In addition, no anti-SARS-CoV-2 nucleocapsid (N) protein IgG antibody was detected in any of the vaccinated or healthy participants, indicating that the anti-SARS-CoV-2 S1 IgG assay is specific for the mRNA vaccine-induced antibodies. The S1 IgG levels detected in fingerstick samples correlated with the levels found in venous blood plasma samples and with the efficacy of venous blood plasma samples in the plaque reduction neutralization test (PRNT). The assay displayed a limit of quantification (LOQ) of 0.59 μg/mL and was found to be linear in the range of 0.51-1000 μg/mL. Finally, its clinical performance displayed a Positive Percent Agreement (PPA) of 100% (95% CI: 0.89-1.00) and a Negative Percent Agreement (NPA) of 100% (95% CI: 0.93-1.00). In summary, the assay described here represents a sensitive, precise, accurate, and simple method for the quantitative detection and monitoring of post-vaccination anti-SARS-CoV-2 spike IgG responses.

## Introduction

COVID-19, which results from infection with the novel severe acute respiratory syndrome coronavirus-2 (SARS-CoV-2), is associated with a spectrum of clinical manifestations ranging from asymptomatic infection to minor flu-like illness to acute respiratory distress syndrome to severe pneumonia and death. SARS-CoV-2 is a single-stranded RNA virus with high sequence identity to SARS-CoV. While worldwide vaccination efforts are ongoing, the COVID-19 pandemic is continuing to spread with 271 million infections, 5.3 million deaths to date and 8337 million vaccine doses administered (Dec.16, 2021) [1].

Antibodies that bind to SARS-CoV-2 spike (S1) protein have shown the potential for blocking viral entry into cells *in vitro* and appear to play a key role in inducing protective immune responses to SARS-CoV-2 infection [2–6]. As a result, a number of COVID-19 vaccines have been developed against the S1 protein and have been administered to hundreds of millions of people in the United States alone. Among the FDA-approved COVID-19 vaccines, the Pfizer BioNTech (BNT162b1 and BNT162b2) and Moderna vaccines (mRNA-1273) are lipid nanoparticle–encapsulated mRNA vaccines directed against the S1 protein, while the Janssen vaccine is an adenovirus (AD26) vector-based vaccine [7]. The time course of antibody responses to vaccinations has shown a gradual increase of anti-SARS-CoV-2 S1 protein IgG levels after the first dose of vaccination followed by a rapid response after the second dose [8]^.^ In similar studies conducted on the Astra Zeneca AZD1222, an adenovirus-vectored vaccine targeting the S1 protein (formerly ChAdOx1 nCoV-19), Folegatti et al reported that anti-spike IgG responses rose by day 28 and were boosted following a second dose. In addition, neutralizing antibody responses against SARS-CoV-2 were detected in 100% participants when measured in the plaque reduction neutralization test titre (PRNT50). In addition, these neutralizing antibody responses correlated with anti-spike IgG responses as measured by ELISA [9].

Additional studies have suggested that a standardized ELISA-based antibody test might be sufficient to predict protection following vaccination [10]. Richmond et al [11] confirmed correlations between results from binding/blocking IgG antibodies as measured by ELISA and neutralizing antibodies as measured by a microneutralization assay in patient samples after a spike (S1)-protein (S-Trimer)-based vaccination. Indeed, consistent with the high IgG and neutralizing antibody titres, a single dose of the S1 protein receptor binding domain (RBD) mRNA conferred near-complete protection against SARS-CoV-2 infection in *hACE2* transgenic mice [12]. In patients infected with SARS-CoV-2, the IgG antibodies to SARS-CoV-2 S1 protein RBD were strongly correlated with neutralizing antibody titres. These titres demonstrated little to no decrease over 75 days after symptom onset in COVID-19 patients [13].

In general, a number of studies on the effectiveness of the Pfizer-BioNTech’s BNT162b2, Moderna’s mRNA-1273 vaccines [14] and the BNT162b2 mRNA vaccine [15] have indicated that anti-SARS-CoV-2 S1 -specific IgG antibodies as measured by ELISA could be used to predict protection after SARS-CoV-2 infection and/or COVID-19 vaccination status. However, despite its precision and accuracy, standard ELISAs require a blood sample collected by venipuncture.

As more vaccines become available for large-scale vaccination, it is highly desirable to have a simple and convenient test for detecting anti-SARS-CoV-2 S1 protein IgG antibody responses. Recently, Karp et al [16] used a finger-prick dried blood spot-based SARS-CoV-2 antibody assay and demonstrated that the results of dried fingerstick blood correlated well with that of paired plasma samples. Iyer et al [13] further confirmed that anti-S1 protein RBD IgG dried blood spots (DBS) measurements had a high degree of linear correlation with plasma samples from the same individuals. In this study, we developed and evaluated the feasibility of using a fingerstick blood sample collected using nylon flocked swabs for the quantitative detection of anti-SARS-CoV-2 S1 protein IgG antibody on a platform reported previously [17]. Our results demonstrated that this type of fingerstick blood sample is sufficient for accurate quantitative measurement of antibody responses after receiving an mRNA COVID-19 vaccine.

## Materials and methods

### Fingerstick blood study design and ethics

Deidentified fingerstick blood samples were used in the study. All fingerstick blood specimens were tested at the DiaCarta Inc. CLIA-certified clinical laboratory. Pre-vaccine and post-vaccine samples were collected from DiaCarta Inc. employees and students from Franciscan University of Steubenville. In addition, fingerstick blood samples from asymptomatic Covid patients, Covid-recovered patients, and healthy unvaccinated persons were collected by the Covid-response team at Franciscan University of Steubenville. Other than anti-SARS-CoV-2 S1 IgG, anti-SARS-CoV-2 N IgG results, vaccination status, and Covid infection status, no other personal information was included in study analysis. This study was approved by the Institutional Review Board (IRB) at Franciscan University of Steubenville (IRB #2021-05). All research was performed in accordance with relevant guidelines and regulations. Informed written consent was obtained from all participants.

### Fingerstick blood dry swab collection

The QuantiVirus^TM^ Fingerstick Blood Collection kit contains disposable lancets, alcohol pads, a swab, a 2.0 mL screw cap tube, and an instruction sheet. The collection procedure is described in Supplementary Figure 1. Briefly, following a fingerstick, blood was collected with a nylon flocked swab (Kangjian, Jiangsu, China) until the swab tip was saturated. The swab was placed into screw cap tube, broken at the score mark, and left in the screw cap tube until further use. The tube was shipped back to the clinical laboratory at DiaCarta, Inc.

To compare the dry swab collection method to more conventional blood collection methods, fingerstick blood samples were collected from some participants in BD Microtainer® MAP microtubes (BD, US, NJ 07417-1880, cat# 363706), and venous blood samples were collected from some participants in K2EDTA tubes containing EDTA as the anticoagulant (BD, cat# 366643). These samples were centrifuged for 10 min at 1600 g at room temperature to collect the plasma for testing.

### SARS-CoV-2 S1 & N protein and reagents

The recombinant SARS-CoV-2 Spike protein S1 (RBD, His Tag) containing 319–541 amino acids of the Spike protein was recombinantly produced in Human Embryonic Kidney cells (Innovative Research, Inc, MI). The SARS-CoV-2 nucleocapsid (N) protein (PMC 827, NP2) was produced using suspension cells (ProMab Biotechnologies Inc, CA). Anti-SARS-CoV-2 Spike S1 Antibody (human chimeric, IgG isotype) was purchased from GenScript Biotech Corporation (Piscataway, NJ). PE-conjugated anti-human IgG Fc antibody was purchased from BioLegend (San Diego, CA). MagPlex Microsphere (magnetic beads) were purchased from Luminex (Austin, TX).

### SARS-CoV-2 S1 & N protein bead conjugation procedure

MagPlex Microsphere and xMAP® Antibody Coupling (AbC) kits were purchased from Luminex (Austin, TX). Recombinant spike protein 1 (S1) RBD was covalently coupled to the surface of MagPlex® Microspheres via a carbodiimide linkage using the xMAP® Antibody Coupling (AbC) kit following the manufacturer’s recommended protocol. Briefly, the stock microspheres were washed and resuspended in Activation Buffer and then combined with the Sulfo-NHS solution and the EDC solution in the bead reaction tube. Following a 20 min incubation, the beads were washed and either the S1 or the N protein was added separately and allowed to incubate for 2 hrs. Following a final wash, the conjugated beads were ready to use for the assay.

### Preparation of assay calibration standards curve

SARS-CoV-2 S1 Antibody (HC2001), produced in cell culture conditions free from animal-derived components was purchased from GenScript Biotech Corporation. The antibody is specific for the SARS-CoV-2 Spike Protein S1 subunit and its RBD domain. To generate the standard curve, HC2001 was serially diluted with PBS-1% BSA buffer to prepare eight standards with various concentrations: 1000, 640, 160, 40, 10, 2.5, 0.62, 0.16 µg/mL.

### Detection of anti-SARS-CoV-2 spike IgG from fingerstick blood dried swab

**Figure 1.**
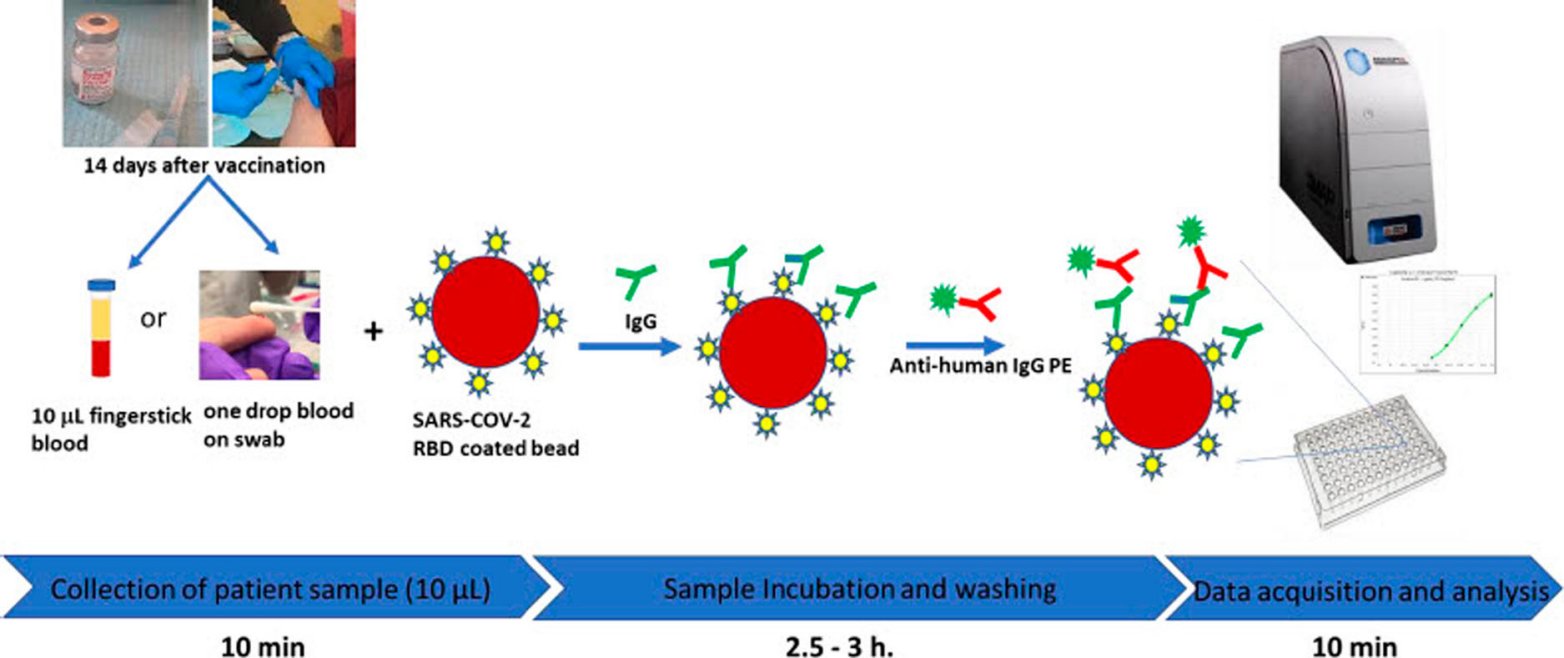
A high-throughput immunoassay platform for anti-SARS-CoV-2 S1 IgG detection using a single drop of fingerstick blood collected in an EDTA tube or on a flocked swab.

**Figure 2.**
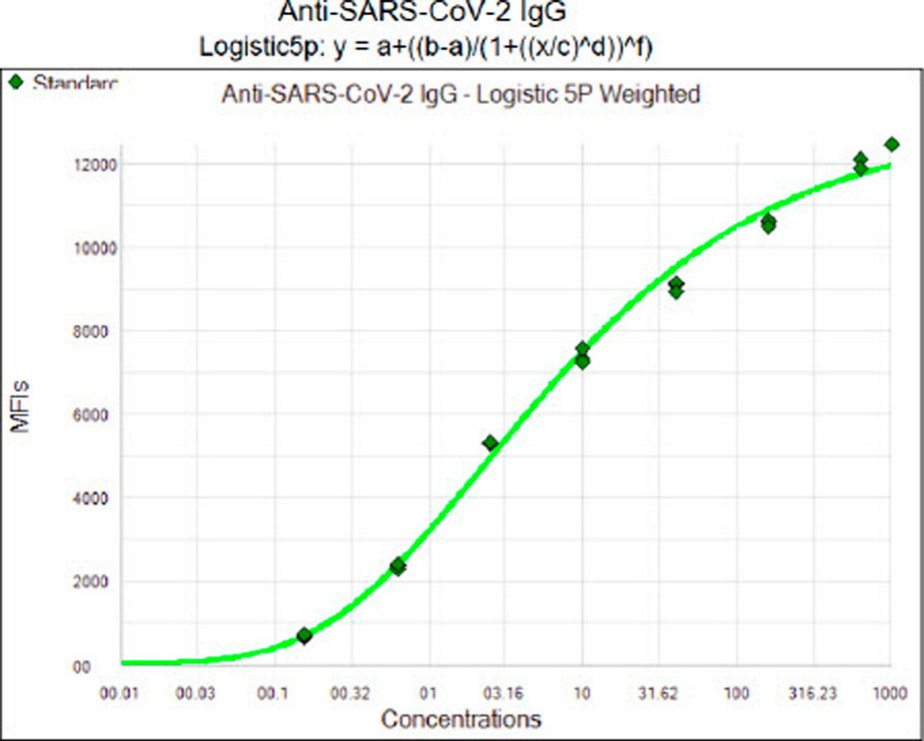
Standard curve generated by Luminex xPONENT software (R^2^ = 0.9996). X-axis: IgG concentration (μg/mL); Y-axis: Median Fluorescence Intensity (MFI).

To analyze the dried blood swab, 300 µL of PBS-1% BSA buffer was added to the 2 mL screw cap tube containing the fingerstick dried blood swab. After incubating for 15 min, the tube was briefly vortexed to release the blood into the buffer and the buffer was then used for IgG testing. The principle of the assay is shown in Figure 1. Recombinant S1 RBD was covalently coupled to the surface of MagPlex® Microspheres (magnetic beads) via a carbodiimide linkage using the xMAP® Antibody Coupling (AbC) kit as described above. S1 RBD protein-coated magnetic beads and blood buffer samples were mixed and incubated at room temperature for 1 h. If IgG antibodies against S1 RBD protein (the antigen) are present in the specimen, they will bind to the S1 RBD protein coated magnetic beads. After washing off unbound proteins, phycoerythrin-(PE) conjugated anti-human IgG antibody was added to the reaction mixture and was incubated at room temperature for 0.5 h. After another washing, PE fluorescence of each well in the 96-well microplate was measured on the MAGPIX® instrument for Median Fluorescence Intensity (MFI). The IgG concentration (μg/mL) was then calculated using the standard curve generated by the Luminex xPONENT software based on the Five Parameter Logistic (5PL) curve fit of the eight standards with various concentrations of IgG: 1000, 640, 160, 40, 10, 2.5, 0.62, 0.16 µg/mL. A quantitative measurement was performed using the Logistic 5P Weighted analysis method on Luminex xPONENT (version 4.2.1705.0) software. A typical standard curve is shown in Figure 2. Using this calibration curve, we were able to calculate the S1 IgG concentration in a specimen from its MFI value as reported previously [17].

### Plaque reduction neutralization test (PRNT)

The PRNT was performed to determine the presence of neutralization antibodies for SARS-CoV-2 in plasma samples at a certified BSL3 laboratory at the University of California, San Francisco. Plasma samples were serially diluted (from 1:100–1:3200) using PBS-0.75% BSA. Diluted plasma samples were inoculated with a fixed number of live SARS-CoV-2 viruses (USA-WA1/2020 from BEI Resources) and incubated at 37°C for 1 hr. The plaque assay was performed by adding the virus and plasma mixture to Vero E6 cells (grown on 12-well plates to 80–90% confluency), followed by 1 hr incubation at 37°C. The estimated number of plaque forming units was 40 per well. After adding Avicel overlay, the plates were incubated at 37°C for 72 h. The cells were then fixed with formalin and stained with crystal violet. The presence of plaques indicates the presence of infected Vero cells in local areas. The plaques in each well were counted, and plaque values for each sample were analyzed in GraphPad Prism to determine the plasma dilution or titre at which the input virus-induced plaques were reduced by 50% (PRNT50). Assay controls used for the PRNT assay include universal negative serum (with viruses), viruses only (without plasma or serum), and culture media only (without viruses).

### Analytical evaluation of the anti-SARS-CoV-2 S1 protein IgG antibody test

#### Analytical performance

a) Limit of Blank (LoB)

LoB was evaluated by testing negative serum samples collected prior to December 2019 in triplicates using two lots of reagents; the measurements were performed on three days. The 95th percentile of the 90 blank measurements is the LoB estimate as per CLSI EP17-A recommendation. Supplementary Table 1a & 1b displays the test results from two lots of reagents. The mean and standard deviation of the blanks samples were found to be 0.03 and 0.03 µg/mL, respectively, with the LoB determined to be 0.08 µg/mL.

b) Limit of Detection (LoD)

According to CLSI EP17-A recommendation, LoD was evaluated by testing five anti-SARS-CoV-2 S1 IgG positive serum samples diluted with negative serum samples collected prior to December 2019 in triplicate using two lots of reagents. The measurements were performed on three separate days. Supplementary Table 2a and Table 2b show the test results from two lots of reagents. The pooled standard deviation of the low level samples was found to be 0.05 µg/mL with the LoD determined to be 0.17 µg/mL.

c) Limit of Quantitation (LoQ)

The analytical LoQ was evaluated by testing the five lowest concentration calibrators in triplicate using two lots of reagents. The measurements were performed on three separate days. Supplementary Table 3a and Table 3b display the test results from two lots of reagents. The analytical LoQ was found to be 0.16 µg/mL with a total error of 0.05 µg/mL at that level. Functional LoQ was estimated using the LoB and the precision of low-level samples and was found to be 0.59 µg/mL.

d) Linearity

The linearity of the test was evaluated following CLSI EP6-A recommendations. Two high-concentration anti-SARS-CoV-2 S1 IgG positive serum samples were serially diluted with different anti-SARS-CoV-2 IgG negative human serum samples and were tested in four replicates. A total of six levels were tested for each sample. The results are shown in Supplementary Table 4. Mean Fluorescent Intensity (MFI) was reported as not all the samples were within the range of the calibrators. Linearity was demonstrated for the interval of 2448–12000 MFI (0.51–1000 µg/mL). Taking into consideration the estimates of LoB, LoD, LoQ, precision, and linearity, the analytical measurement range of the assay was determined to span 0.59–1000 µg/mL.

e) Precision

Within-run precision (repeatability) was evaluated by testing negative samples and positive samples in 21 or 24 replicates. Between-run precision was evaluated by testing a negative sample and a positive sample on five separate runs (3∼4 replicates per run). As shown in SupplementaryTable 5a and Table 5b, the average CV% of within-run precision and between-run precision was 8.25% (from 4.71% to 11.74%) and 10.47% (from 5.0% to 13.4%), respectively.

f) Interference

Hemoglobin (200 mg/dL) was spiked into two negatives and two positive serum samples to test the potential interfering effect of high levels of hemoglobin, which might be present following hemolysis and other conditions. Similarly, EDTA (10mM) was spiked into two negative and two positive serum samples to test the potential interfering effect of EDTA which is the anticoagulant used in EDTA blood collection tubes. As shown in Supplementary Table 6, no difference in the fluorescence signal (MFI) was observed between Control and EDTA-spike samples (t-test, *p* > 0.05). For hemoglobin, with positive serum #1, although a significant difference was observed in MFI between Control and Hemoglobin-spike samples (*p* = 0.01), the difference was only 2.85%, well within the average CV% of within-run precision. Therefore, both EDTA and hemoglobin do not have a significant interfering effect on the anti-SARS-CoV-2 S1 IgG assay at the tested concentrations.

### Stability of fingerstick blood in dried swabs

To test the stability of fingerstick blood collected using nylon flocked swabs and stored at room temperature (20 ± 5°C), a total 7 of dried swab samples were tested for signal intensity on day 1, 2, and 4 at room temperature. As shown in Supplementary Table 7a, the samples were stable for up to 4 days at room temperature.

To further confirm its stability during shipping, dried swab samples were collected in triplicate from 10 different vaccinated individuals. These samples were shipped overnight from Ohio to the DiaCarta CLIA laboratory in California. The samples were kept at room temperature (20 ± 5°C) for 7 days. As shown in Supplementary Table 7b, dried swab samples were stable for up to 7 days following shipping.

### Clinical performance evaluation of the anti-SARS-CoV-2 S1 protein IgG antibody test

31 whole blood samples were collected from individuals that had previously confirmed SARS-CoV-2 infection as determined by an FDA-authorized SARS-CoV-2 RT–PCR test. In addition, 51 whole blood samples were collected from individuals who never exhibited COVID symptoms and who tested negative (within 1 week of the sample collection) by an FDA EUA authorized RT-qPCR method. The results showed a Positive Percent Agreement (PPA) of 100% (95% CI: 0.89-1.00) and a Negative Percent Agreement (NPA) of 100% (95% CI: 0.93-1.00) (Supplementary Table 8a & 8**b**). Based upon these results, the clinical sensitivity of the test was 100% (95% CI: 0.863-1.00) and its specificity was 100% (95% CI: 0.913-1.00).

## Results

### Anti-SARS-CoV-2 S1 IgG antibody responses after SARS-CoV-2 mRNA vaccine

Previously, we reported the development of a semi-quantitative immunoassay for anti-SARS-CoV-2 IgG^18^, which showed a positive percent agreement (PPA) of 46.15%, 61.54%, and 97.53% for samples collected in time periods of 0–7 days, 8–14 days, and ≥15 days, respectively, from symptom onset in confirmed COVID-19 subjects, and a negative percent agreement (NPA) of 98.23%. In this study, we aimed to develop a quantitative immunoassay using fingerstick blood samples to evaluate post-vaccination immune responses for SARS-CoV-2.

Since venous blood sampling requires venipuncture and could be logistically challenging to collect and process during the ongoing pandemic, we evaluated the feasibility of at-home collection of fingerstick blood, followed by shipment to a central laboratory for anti-SARS-CoV-2 S1 IgG antibody detection.

**Figure 3.**
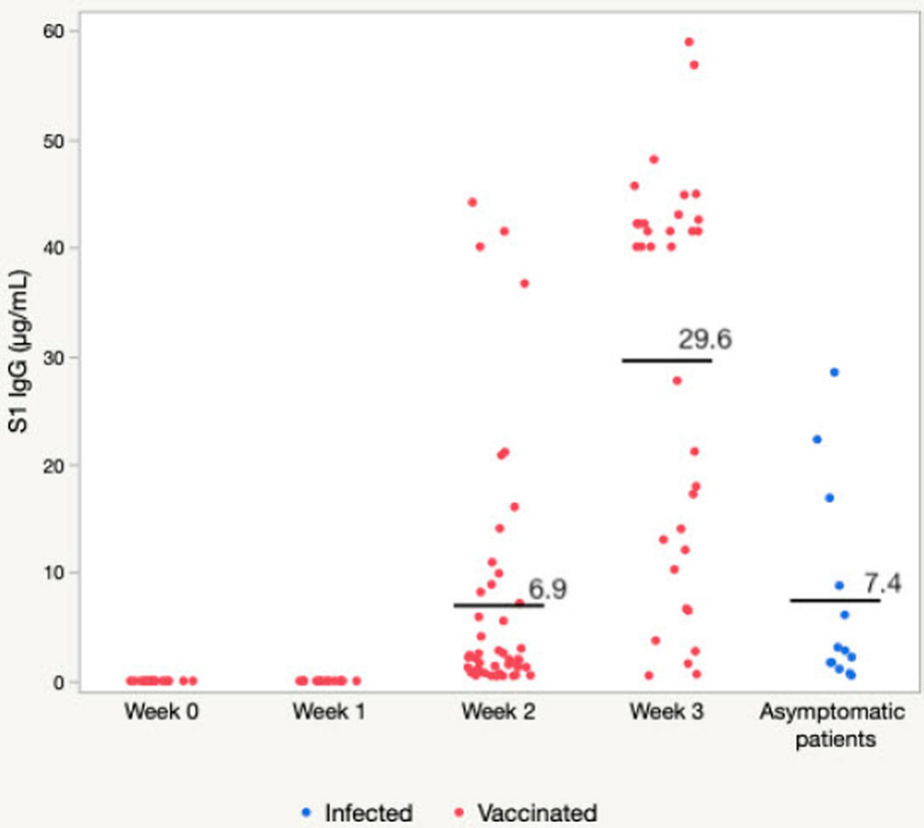
Quantitative measurements of anti-SARS-CoV-2 spike IgG before and after receiving the first dose of the Moderna COVID-19 mRNA vaccine. The floating lines and numbers represent the mean values for each sample group. Week 0-1, N = 53; Week 2, N = 38; Week 3, N = 44.

We quantitatively analyzed anti-SARS-CoV-2 S1 IgG in fingerstick blood collected from 53 individuals without a history of COVID-19 as confirmed by an FDA-EUA authorized RT–PCR kit (QuantiVirus^TM^ SARS-CoV-2 Test Kit, DiaCarta Inc, CA). As shown in Figure 3, no anti-SARS-CoV-2 S1 IgG antibody was detected before vaccination or on day 7 post first dose of vaccine in this population. Two weeks after the first dose of vaccine, anti-SARS-CoV-2 S1 IgG detection rate was 92.1% in those individual samples (Table 1). Interestingly, all 3 negative test results were observed in individuals over 80 years of age. By three weeks after the first dose of vaccination, the mean value of the IgG levels detected increased remarkably (Figure 3). In addition, by three weeks all the subjects tested positive for anti-SARS-CoV-2 S1 IgG (a detection rate of 100%, Table 1). The lowest level detected was 2.74 µg/mL, while the highest levels detected were 59.01 µg/mL, with a mean of 29.6 µg/mL of IgG antibody after three weeks. This data indicates that a dried fingerstick blood sample is sufficient for detecting and monitoring the development of anti-SARS-CoV-2 Spike IgG antibody after vaccination.

**Table 1.** Fingerstick blood-based testing of anti-SARS-CoV-2S1 IgG after COVID-19 vaccination.

Time of Vaccination	Number of Subjects	IgG Positive Detection Rate
Pre-vaccination	42	0 (0%)
1 week post 1st dose vaccination	53	0 (0%)
2 weeks post 1st dose vaccination	38	35 (92.1%)
3 weeks post 1st dose vaccination	44	44 (100%)
1 week post 2nd dose vaccination	45	45 (100%)

**Figure 4.**
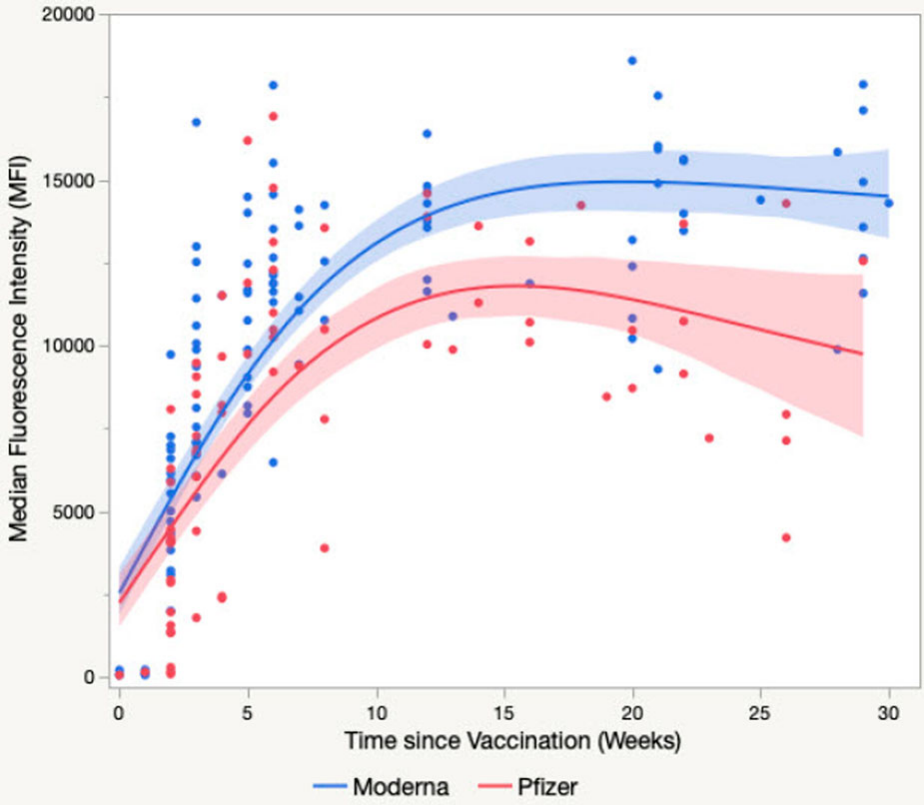
SARS-CoV-2 mRNA vaccine-induced humoral immune responses. Shown are the semi-quantitative anti-SARS-CoV-2 S1 IgG antibody levels from 76 vaccinated participants (213 data points). *P *= 0.198 from Week 0 to Week 12; *P < *0.001 after Week 13 using the two-tailed Mann–Whitney U test.

Not surprisingly, the second dose was able boost the anti-SARS-CoV-2 S1 antibody levels considerably from week 5 to week 7 (week 4 was the timepoint for the second vaccine dose) (Figure 4**).** Additional participants were recruited for this longer-term analysis (76 participants total with 213 data points.) The second dose boosted the S1 IgG levels much higher than the 1000 μg/mL cut off level for the linearity of the assay. As a result, the raw MFI values are presented for these samples as a semi-quantitative measure of S1 IgG levels. Using these semi-quantitative values, the average IgG concentration was found to increase gradually after the second dose and attain the highest level three months post-vaccination. The levels remained stable five months after vaccination and then decreased slightly seven months post vaccination (Figure 4). After 13 weeks, SARS-CoV-2 mRNA vaccine-induced humoral immune responses did show a significant difference in S1 IgG levels between Moderna and Pfizer vaccines (*P *= 0.001) using a two-tailed Mann–Whitney U test (Figure 4).

### Comparison of fingerstick blood samples collected in an EDTA tube versus on a dried swab

**Figure 5.**
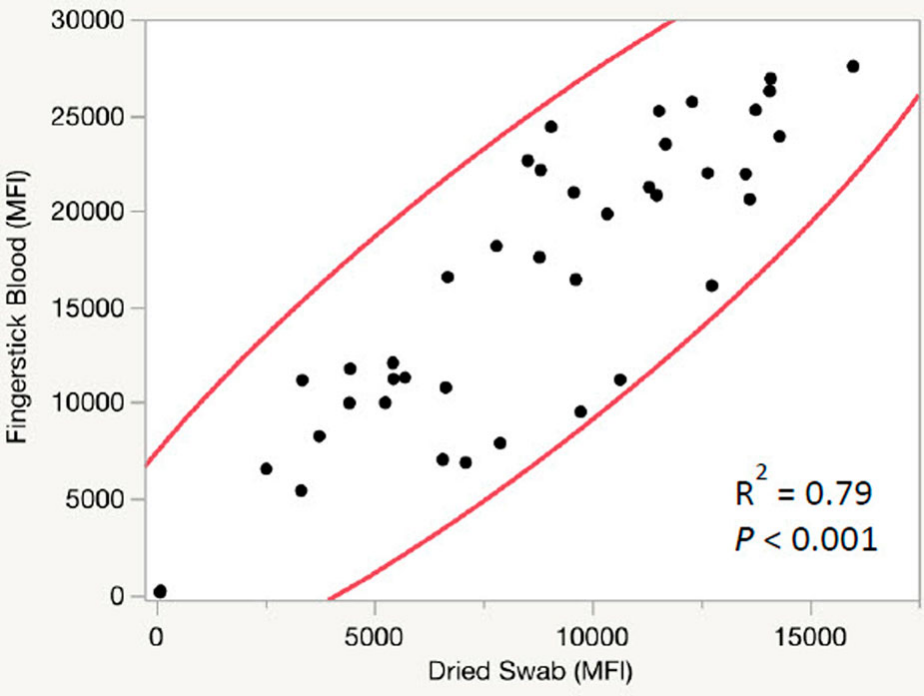
Comparison of MFI from fingerstick blood samples collected in EDTA tubes vs those collected on dried swabs. Bivariate Normal Ellipse *P* = 0.95. Number of samples = 43.

We examined the relationship between S1 IgG levels detected in fingertip blood samples stored in an EDTA tube or on a dried flocked swab. Forty-three paired fingerstick blood samples from vaccinated donors were compared side by side. Overall, the samples in the EDTA tubes showed higher MFI signals than the dried swab samples. Nonetheless, the signals are highly correlated between the two sample types with an R^2^ of 0.79 and *P *< 0.001 (Figure 5).

### Comparison of venous plasma samples vs fingerstick blood dried swab samples

**Figure 6.**
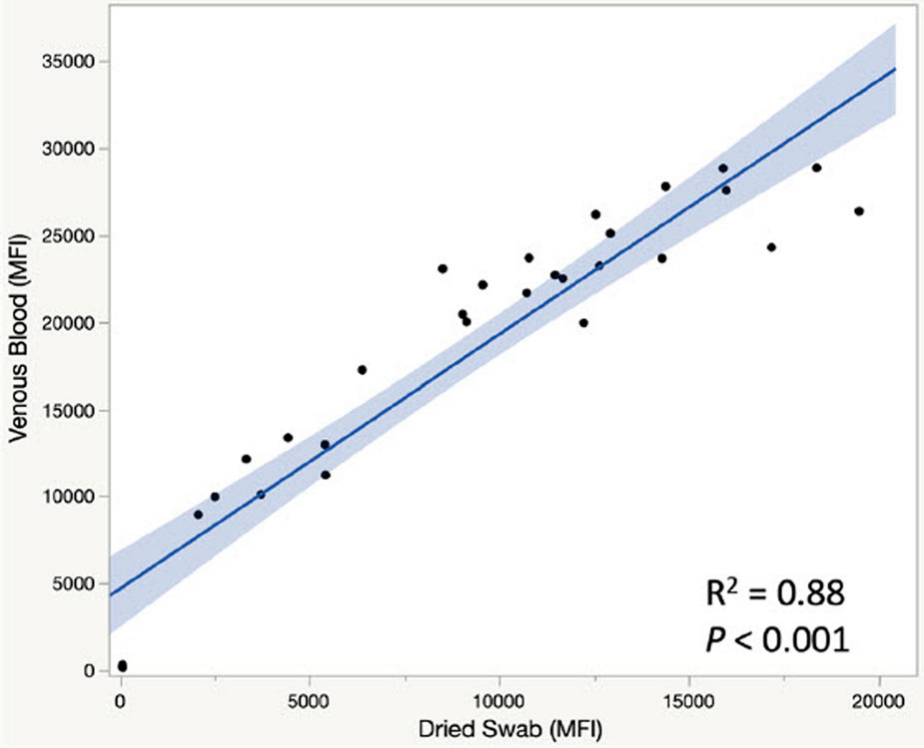
Comparison of MFI between venous blood plasma in EDTA tube and fingerstick blood on dried swab. R^2 ^= 0.88, and *P*-value for the coefficient of dried swab < 0.001. Number of samples  = 31.

We then compared pairs of venous plasma samples and dried swab samples collected from the same vaccinated donors. A side-by-side comparison of paired samples from thirty-one vaccinated donors is shown in Figure 6. On average, the MFI values in plasma samples were about 2.3 times higher than that of fingerstick blood dried swab samples. The higher signals in plasma samples are expected as plasma obtained from venous blood contains a higher concentration of IgG antibodies than the more diluted fingerstick whole blood. Nonetheless, a significant correlation was observed between venous plasma samples and fingerstick blood dried swabs (R^2  ^= 0.88, and *P* <0.001, Figure 6).

### PRNT-based neutralizing antibody activity

**Figure 7.**
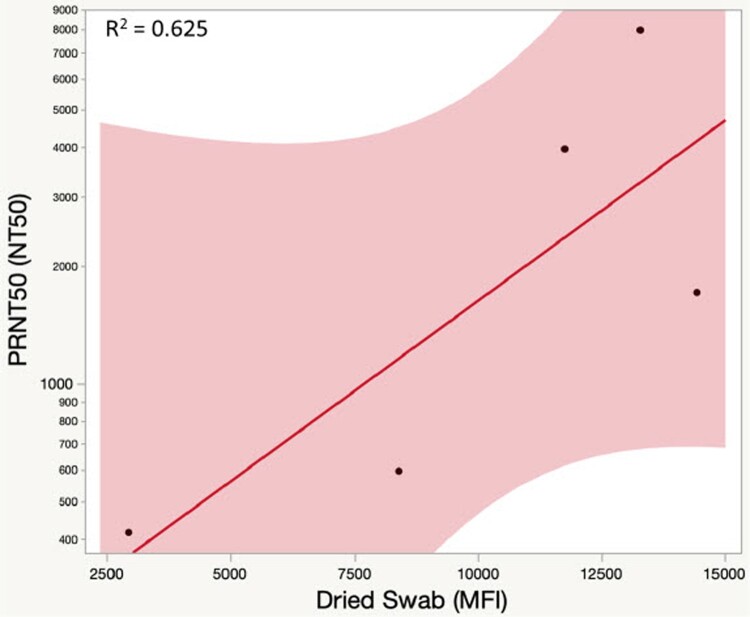
Microsphere-based anti-SARS-CoV-2 S1 IgG versus PRNT-based neutralizing antibody levels. Linear fit on semi-log plot, R^2 ^= 0.625.

To determine whether the detection of anti-SARS-CoV-2 S1 IgG antibody in plasma or fingerstick blood samples predicts protective immunity, cell culture-based plaque reduction neutralization test (PRNT) using live SARS-CoV-2 virus was performed. Due to capacity limitation, only 5 plasma samples were tested, and the results are listed in Supplementary Table 9. These samples were collected from donors who had been fully vaccinated (4 received Moderna vaccine, 1 received Pfizer vaccine), 2 or more weeks after the second vaccine dose. As shown in Supplementary Table 9, all 5 samples demonstrated strong SARS-CoV-2 neutralizing activity, with PRNT50 values ranging from 415 to 7983. The fingerstick swab S1 IgG antibody levels (MFI values) correlated with the neutralizing antibody activities (PRNT50 values) of the corresponding blood plasma with R^2  ^= 0.625 using a linear fit model on a semi-log plot (Figure 7).

### mRNA vaccine-elicited anti-SARS-CoV-2 S1 IgG can be distinguished from anti-SARS-CoV-2 N protein IgG caused by infection

**Figure 8.**
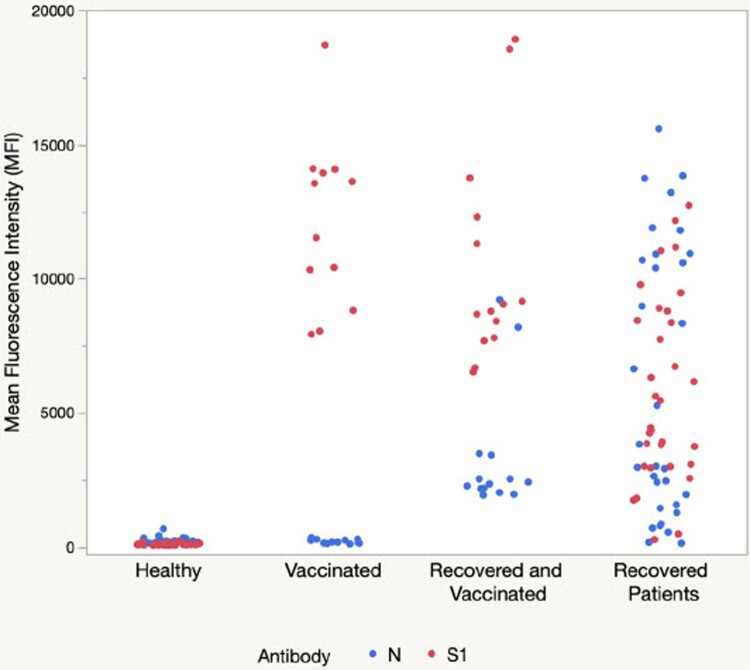
Comparison of anti-SARS-CoV-2 S1 IgG and anti-SARS-CoV-2 N protein IgG in vaccinated and previously infected groups. Fingerstick blood sample-based antibody immunoassay using SARS-CoV-2 S1 RBD protein and nucleocapsid (N) protein-coupled microspheres.

To confirm that the reported MFI signals were indeed specific for the SARS-CoV-2 S1 protein IgG antibodies elicited by the mRNA vaccine, additional blood samples were collected and tested using microspheres conjugated with SARS-CoV-2 nucleocapsid protein (N protein) and SARS-CoV-2 S1 RBD protein-coupled microspheres separately. A total 103 samples were tested, including 32 samples collected from recovered COVID-19 patients, 14 from recovered and vaccinated COVID-19 patients, 17 from vaccinated healthy individuals, and 40 samples from healthy non-vaccinated individuals. As shown in Figure 8, for samples collected from the vaccinated healthy individuals, SARS-CoV-2 S1 RBD protein-coupled microspheres detected a high signal with an average MFI of 12,086, whereas the signal generated by N protein-coupled microspheres was close to the background about 208 MFI. The MFI signals for both anti-S1 IgG and anti-N protein IgG were close to baseline levels in the group of non-vaccinated healthy individuals. In addition, the samples from the non-vaccinated COVID-19 recovered patients had comparable levels of anti-S1 IgG and anti-N protein IgG, whereas the samples from the vaccinated COVID-19 recovered individuals showed significantly higher anti-S1 IgG (mean 10541 MFI) than anti-N protein IgG (mean 3337 MFI). These results suggest that COVID-19 recovered individuals, vaccinated individuals, and COVID-19 recovered plus vaccinated individuals could all be distinguished from each other by detecting levels of both anti-S1 IgG and anti-N IgG simultaneously.

## Discussion

The data presented here demonstrate the efficacy of an easy-to-use fingerstick blood collection method for detecting and monitoring anti-SARS-CoV-2 S1 IgG after full COVID-19 vaccination. The results from fingerstick blood collected on a flocked dry swab are comparable to fingerstick blood collected into an EDTA tube for anti-SARS-CoV-2 S1 IgG detection following vaccination. In addition, the fingerstick blood collected on a dried swab also showed a high correlation with the anti-SARS-CoV-2 S1 IgG levels found in venous plasma samples. This approach is more convenient for end users as it does not require venipuncture or a visit to a healthcare facility or a diagnostic laboratory. Furthermore, we have also shown that the fingerstick blood can be successfully applied to a quantitative anti-SARS-CoV-2 S1 IgG assay, which can be used for monitoring the antibody response status over time after full vaccination and/or boosters with COVID mRNA vaccines.

Our study indicated that 2 weeks after receiving the first dose of mRNA vaccine, a significant amount of anti-SARS-CoV-2 S1 IgG antibody can be detected in most individuals (with the exception of three individuals who were all over 80 years old) at an average level of 6.9 µg/mL. This result is consistent with a recent report, which showed that the antibody response increased up to 1016 AUC on days 13–16, compared to 1 AUC on 0–4 days after receiving the vaccine, as determined by ELISA [8]. In addition, the data also demonstrated that COVID-19 mRNA vaccines appeared to be more efficient in inducing S1 IgG antibody responses than cases of mild asymptomatic SARS-CoV-2 infection (Figure 3).

These results demonstrated that the fingerstick blood-based testing method is sufficient for quantitatively detecting and monitoring the development of anti-SARS-CoV-2 S1 IgG antibody after vaccination. Furthermore, consistent with previous reports [18–21], on week 3 post vaccination, the mean value of S1 IgG increased remarkably. In fact, the second dose boosted the IgG level much higher than the 1000 μg/mL limit for the liner range in this assay. While the data for these samples is reported semi-quantitatively, a quantitative S1 IgG measurement could easily be obtained in the future by a further dilution of these samples.

The assay described here is specific for the vaccine-elicited anti-SARS-CoV-2 S1 IgG antibody and is not reactive to anti-SARS-CoV-2 nucleocapsid (N) IgG antibody. When combined with SARS-CoV-2 N protein-coupled microspheres, the assay can be used to distinguish vaccinated individuals from those with a prior infection as the SARS-CoV-2 S1 mRNA vaccines stimulate only an anti-SARS-CoV-2 spike IgG antibody response and not an anti-SARS-CoV-2 N protein IgG antibody response. In addition, we observed that anti-S1 IgG and anti-N protein IgG levels were similar in COVID-19 recovered individuals, whereas anti-S1 IgG levels were higher than anti-N protein IgG in COVID-19 recovered individuals who had also been vaccinated. Therefore, this test could be useful for identifying vaccinated individuals vs COVID-19 recovered individuals vs COVID-19 recovered + vaccinated individuals if the sample is simultaneously tested for both anti-S1 and anti-N antibodies.

The assay was very specific as we observed no detectable anti-SARS-CoV-2 S1 IgG before vaccination or during the first week post-vaccination among individuals who had not been previously diagnosed with COVID-19 infection. In general. the antibody became detectable 2 weeks after the first dose, averaging 5000 MFI and increased to about 12,000–13,000 MFI by 7–8 weeks (i.e. 3–4 weeks after second vaccine dose, Figure 4). Interestingly, one individual infected with COVID-19 in 2020 showed IgG signal intensity that increased from 1,300 MFI to over 15,000 MFI within 1 week after the first dose of vaccination in early 2021 (data not shown here). This is in agreement with previous observations that seropositive individuals have higher responses after the first dose [8,22,23]. Marot et al [24] reported that anti-S1 IgG and anti-S1-RBD IgG antibody levels in previously confirmed COVID-19 patients did not change significantly between 3 weeks and 3 months, despite a slight increase at 2 months. Based on our data, the vaccine induced S1 IgG antibody responses appear to follow the same course, reaching 12,000 MFI to 13,000 MFI 6–8 weeks after receiving vaccine and staying at 14,000 MFI by 15 weeks, followed by gradual decline in levels. Interestingly, Moderna vaccinated individuals on average displayed higher S1 IgG levels than those vaccinated with the Pfizer BioNTech vaccine. This data agrees with a recent report that demonstrated a significantly higher humoral immunogenicity with the SARS-CoV-2 mRNA-1273 vaccine (Moderna) compared to the BNT162b2 vaccine (Pfizer BioNTech) [25].

The neutralization antibody titre will likely be an important predictor of vaccine efficacy in the future as new vaccines are developed [26]. In fact, the SARS-CoV-2 neutralization (PRNT50) assay has demonstrated a significant correlation with antibody levels (including anti-S1-RBD IgG) as measured by immunoassays [27]. Our anti-SARS-CoV-2 S1 IgG data from the fingerstick blood assay also showed a close correlation with the results of the SAES-CoV-2 neutralization assay. Previous studies have found an average PRNT50 NT_50_ value of about 451 for vaccinated people [20]. This is consistent with what we observed, with individuals who had fingerstick blood IgG levels of 2,944 MFI and 8,408 MFI showing PRNT50 NT_50_ values of 415 and 596, respectively. Given the fact that after the first dose, vaccinated people displayed an average IgG of 4,000 -5,000 MFI, we estimate that they should have PRNT50 NT_50_ values above 451. Taken together, high levels of anti-SARS-CoV-2 spike IgG antibodies detected by immunoassays could indicate protective immunity against SARS-COV-2.

As mentioned above, all 3 samples in which anti-SARS-CoV-2 S1 IgG was undetectable two weeks after vaccination were from individuals over 80 years of age old. This observation suggests that not all vaccinated people will have detectable humoral immune responses within 2 weeks of the first vaccine dose, especially in the elderly population. However, all voluntary subjects, including the elderly, developed robust IgG antibody responses after the second dose of mRNA vaccine. It should be noted that none of the voluntary subjects were considered immunocompromised.

In conclusion, we demonstrated that dried fingerstick blood collected onto flocked swab can be used for the quantitative detection and monitoring of anti-SARS-CoV-2 Spike IgG responses after COVID-19 vaccination. This methodology is appealing because it makes it possible for vaccinated individuals to collect samples at home for subsequent testing without visiting healthcare facilities or clinical laboratories.

## Supplementary Material

Supplemental Material
