# Supplementary material for: A new testing platform using fingerstick blood for quantitative antibody response evaluation after SARS-CoV-2 vaccination
Source: Emerg Microbes Infect. 2022 Jan 7;11(1):250–9. doi: 10.1080/22221751.2021.2023328 (PMC8745372; doi:10.1080/22221751.2021.2023328)
Supplement: Supplemental Material [file TEMI_A_2023328_SM0134.docx]

**Supplementary Information**

**A New Testing Platform Using Fingerstick Blood for Quantitative Antibody Response Evaluation after SARS-CoV-2 Vaccination**

Jinwei Du^1#^, Dayu Zhang^1#^, Joseph A. Pathakamuri^2^, Daniel Kuebler^2^, Ying Yang^1^, Yulia Loginova^1^, Eric Chu^1^, Roberta Madej^1^, Jocelyn V. Neves^2^, Brianna Singer^2^, Holly Radke^2^, Naomi Spencer^2^, Elizabeth Rizk^2^, Aiguo Zhang^1^, Chuanyi M. Lu^3^ and Michael Y. Sha^1^*

^1^DiaCarta Inc., 4385 Hopyard Rd. suite 100, Pleasanton, CA 94588, US. ^2^ Franciscan University of Steubenville, Steubenville, OH. 43952. ^3^ University of California and VA Health Care System, San Francisco, CA.

# These authors contributed equally

*****Correspondence: [msha@diacarta.com](mailto:msha@diacarta.com)


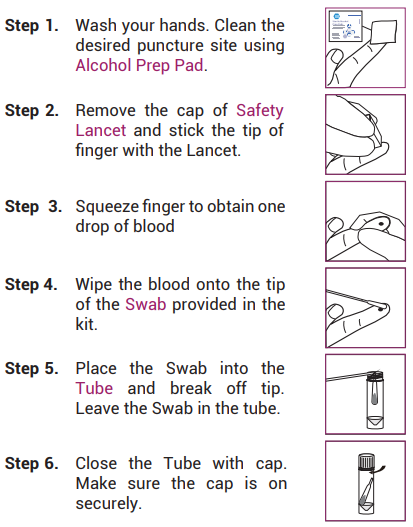


**Supplementary Figure 1**. Fingerstick blood collection procedure.

**Supplementary Table 1a**. LoB results from Lot-1

| IgG Test | Day 1 (µg/mL) | | | Day 2 (µg/mL) | | | Day3 (µg/mL) | | |
| --- | --- | --- | --- | --- | --- | --- | --- | --- | --- |
| Sample | Test-1 | Test-2 | Test-3 | Test-1 | Test-2 | Test-3 | Test-1 | Test-2 | Test-3 |
| B1 | 0.030 | 0.036 | 0.035 | 0.031 | 0.030 | 0.031 | 0.025 | 0.025 | 0.023 |
| B2 | 0.042 | 0.039 | 0.041 | 0.039 | 0.037 | 0.039 | 0.026 | 0.024 | 0.027 |
| B3 | 0.031 | 0.031 | 0.029 | 0.026 | 0.031 | 0.026 | 0.021 | 0.020 | 0.022 |
| B4 | 0.035 | 0.031 | 0.029 | 0.028 | 0.026 | 0.033 | 0.022 | 0.026 | 0.024 |
| B5 | 0.045 | 0.034 | 0.041 | 0.036 | 0.037 | 0.036 | 0.028 | 0.022 | 0.029 |

* The results are anti-SARS-CoV-2 IgG concentrations in (µg/mL)

**Supplementary Table 1b**. LoB results from Lot-2

| IgG Test | Day 1 (µg/mL) | | | Day 2 (µg/mL) | | | Day3 (µg/mL) | | |
| --- | --- | --- | --- | --- | --- | --- | --- | --- | --- |
| Sample | Test-1 | Test-2 | Test-3 | Test-1 | Test-2 | Test-3 | Test-1 | Test-2 | Test-3 |
| B1 | 0.016 | 0.012 | 0.013 | 0.026 | 0.024 | 0.023 | 0.019 | 0.017 | 0.171 |
| B2 | 0.022 | 0.019 | 0.014 | 0.031 | 0.030 | 0.028 | 0.023 | 0.021 | 0.294 |
| B3 | 0.012 | 0.013 | 0.013 | N/A | 0.025 | 0.027 | 0.013 | 0.016 | 0.013 |
| B4 | 0.013 | 0.024 | 0.018 | 0.025 | 0.026 | 0.026 | 0.022 | 0.017 | 0.016 |
| B5 | 0.019 | 0.015 | 0.014 | 0.024 | 0.026 | 0.026 | 0.020 | 0.023 | 0.018 |

*The results are anti-SARS-CoV-2 IgG concentrations in (µg/mL)

**Supplementary Table 2a** LoD results from Lot-1

| IgG Test | Day 1 (µg/mL) | | | Day 2 (µg/mL) | | | Day3 (µg/mL) | | |
| --- | --- | --- | --- | --- | --- | --- | --- | --- | --- |
| Sample | Test-1 | Test-2 | Test-3 | Test-1 | Test-2 | Test-3 | Test-1 | Test-2 | Test-3 |
| D1 | 0.302 | 0.477 | 0.257 | 0.319 | 0.276 | 0.313 | 0.280 | 0.276 | 0.243 |
| D2 | 0.177 | 0.188 | 0.129 | 0.165 | 0.116 | 0.138 | 0.155 | 0.116 | 0.135 |
| D3 | 0.235 | 0.226 | 0.231 | 0.212 | 0.209 | 0.212 | 0.192 | 0.174 | 0.189 |
| D4 | 0.128 | 0.116 | 0.157 | 0.164 | 0.170 | 0.178 | 0.177 | 0.160 | 0.133 |
| D5 | 0.321 | 0.188 | 0.192 | 0.294 | 0.279 | 0.318 | 0.228 | 0.228 | 0.314 |

*The results are anti-SARS-CoV-2 IgG concentrations in µg/mL.

**Supplementary Table 2b**. LoD results from Lot-2

| IgG Test | Day 1 (µg/mL) | | | Day 2 (µg/mL) | | | Day3 (µg/mL) | | |
| --- | --- | --- | --- | --- | --- | --- | --- | --- | --- |
| Sample | Test-1 | Test-2 | Test-3 | Test-1 | Test-2 | Test-3 | Test-1 | Test-2 | Test-3 |
| D1 | 0.448 | 0.206 | 0.169 | 0.422 | 0.319 | 0.386 | 0.359 | 0.237 | 0.215 |
| D2 | 0.088 | 0.082 | 0.122 | 0.172 | 0.143 | 0.197 | 0.092 | 0.148 | 0.095 |
| D3 | 0.132 | 0.199 | 0.108 | 0.210 | 0.205 | 0.219 | 0.172 | 0.144 | 0.164 |
| D4 | 0.211 | 0.157 | 0.146 | 0.196 | 0.149 | 0.192 | 0.171 | 0.108 | 0.153 |
| D5 | 0.188 | 0.305 | 0.249 | 0.250 | 0.304 | 0.188 | 0.294 | 0.245 | 0.266 |

***** The results are anti-SARS-CoV-2 IgG concentrations in µg/mL

**Supplementary Table 3a**. LoQ results from Lot-1

| IgG Test | Day 1 (µg/mL) | | | Day 2 (µg/mL) | | | Day3 (µg/mL) | | |
| --- | --- | --- | --- | --- | --- | --- | --- | --- | --- |
| Sample | Test-1 | Test-2 | Test-3 | Test-1 | Test-2 | Test-3 | Test-1 | Test-2 | Test-3 |
| Q1 | 0.140 | 0.143 | 0.139 | 0.159 | 0.158 | 0.153 | 0.161 | 0.160 | 0.158 |
| Q2 | 0.169 | 0.155 | 0.148 | 0.136 | 0.144 | 0.150 | 0.152 | 0.162 | 0.156 |
| Q3 | 0.149 | 0.142 | 0.139 | 0.156 | 0.151 | 0.164 | 0.153 | 0.159 | 0.157 |
| Q4 | 0.157 | 0.157 | 0.157 | 0.142 | 0.156 | 0.153 | 0.156 | 0.163 | 0.151 |
| Q5 | 0.156 | 0.154 | 0.160 | 0.178 | 0.136 | 0.168 | 0.157 | 0.176 | 0.138 |

*The results are anti-SARS-CoV-2 IgG concentrations in µg/mL.

**Supplementary Table 3b.** LoQ results from Lot-2

| IgG Test | Day 1 (µg/mL) | | | Day 2 (µg/mL) | | | Day3 (µg/mL) | | |
| --- | --- | --- | --- | --- | --- | --- | --- | --- | --- |
| Sample | Test-1 | Test-2 | Test-3 | Test-1 | Test-2 | Test-3 | Test-1 | Test-2 | Test-3 |
| Q1 | 0.156 | 0.155 | 0.160 | 0.159 | 0.158 | 0.152 | 0.158 | 0.157 | 0.157 |
| Q2 | 0.196 | 0.176 | 0.167 | 0.150 | 0.157 | 0.164 | 0.149 | 0.159 | 0.153 |
| Q3 | 0.164 | 0.155 | 0.152 | 0.171 | 0.166 | 0.179 | 0.146 | 0.162 | 0.165 |
| Q4 | 0.173 | 0.173 | 0.168 | 0.148 | 0.163 | 0.161 | 0.153 | 0.154 | 0.163 |
| Q5 | 0.156 | 0.154 | 0.160 | 0.189 | 0.142 | 0.177 | 0.150 | 0.158 | 0.162 |

*The results are anti-SARS-CoV-2 IgG concentrations in µg/mL.

**Supplementary Table 4**. Linearity results from two serially diluted anti-SARS-CoV-2 S1 IgG positive serum samples. Best fit curve of the MFI_Sample_1 is displayed below showing the linear range of the assay. (Curve generated by Prism software (R^2^ = 0.9956).

| Dilution | MFI_Sample_1 | MFI_Sample_2 |
| --- | --- | --- |
| 1 | 11479 | 11362 |
| 2 | 10595 | 9536 |
| 3 | 6322 | 8359 |
| 4 | 2448 | 5613 |
| 5 | 946 | 2340 |
| 6 | 401 | 635 |

**Supplementary Table 5a**. Within-run Precision (repeatability)

| Sample | Number of Replicates | Average MFI | StDev | CV% |
| --- | --- | --- | --- | --- |
| Negative serum sample #1 | 24 | 25 | 2.9 | 11.74% |
| Negative serum sample #2 | 21 | 16 | 0.7 | 4.71% |
| Positive serum sample #1 | 24 | 608 | 65.7 | 10.80% |
| Positive serum sample #2 | 21 | 363 | 20.8 | 5.73% |

**Supplementary Table 5b**. Between-run Precision

| Sample | Test-1 | Test-2 | Test-3 | Test-4 | Test-5 | Average MFI | StDev | CV% |
| --- | --- | --- | --- | --- | --- | --- | --- | --- |
| Blank | 13 | 13 | 12.5 | 14 | 14 | 13.3 | 0.67 | 5.00% |
| Negative sample | 19 | 25 | 18.7 | 23 | 20 | 21.1 | 2.75 | 13.00% |
| Positive sample | 828.9 | 608 | 791.3 | 827 | 673 | 745.6 | 99.82 | 13.40% |

**Supplementary Table 6**. Interfering Effect Evaluation

| Sample | Group | MFI-1 | MFI-2 | MFI-3 | MFI-4 | Average | StDev | CV% | t-test  p value | difference (%) |
| --- | --- | --- | --- | --- | --- | --- | --- | --- | --- | --- |
| Negative serum #1 | Control | 19 | 19 | 19 | 19 | 19.0 | 0.00 | 0.0% |  |  |
|  | EDTA | 18 | 17 | 18 | 18 | 17.8 | 0.40 | 2.4% | 0.19 | 6.6% |
|  | Hemoglobin | 18 | 17 | 17 | 18 | 17.5 | 0.50 | 2.9% | 0.19 | 7.9% |
| Negative serum #2 | Control | 20 | 19 | 22 | n/a | 20.3 | 1.53 | 7.5% |  |  |
|  | EDTA | 14 | 19 | 21 | n/a | 18.0 | 3.61 | 20.0% | 0.17 | -11.5% |
|  | Hemoglobin | 16 | 17 | 21 | n/a | 18.0 | 2.65 | 14.7% | 0.06 | -11.5% |
| Positive serum #1 | Control | 841 | 818 | 842 | 815 | 828.9 | 12.70 | 1.5% |  |  |
|  | EDTA | 802 | 803 | 869 | 814 | 821.9 | 27.30 | 3.3% | 0.32 | 0.8% |
|  | Hemoglobin | 805 | 799 | 824 | 793 | 805.3 | 11.60 | 1.4% | 0.01 | 2.9% |
| Positive serum #2 | Control | 705 | 606 | 629 | n/a | 646.7 | 51.81 | 8.0% |  |  |
|  | EDTA | 704 | 624 | 668 | n/a | 665.3 | 40.07 | 6.0% | 0.12 | 2.9% |
|  | Hemoglobin | 769 | 654 | 640 | n/a | 687.7 | 70.78 | 10.3% | 0.06 | 6.3% |

**Supplementary Table 7a**. Stability evaluation of fingerstick blood dried swab for anti-SARS-CoV-2 IgG immunoassay

| Sample ID | Day 1 | | Day 2 | | Day 4 | |
| --- | --- | --- | --- | --- | --- | --- |
|  | MFI | Interpretation | MFI | Interpretation | MFI | Interpretation |
| Dia161 | 4342 | Positive | 4241 | Positive | n/a | n/a |
| Dia152 | 7771 | Positive | 7533 | Positive | n/a | n/a |
| Dia151 | 11345 | Positive | n/a | n/a | 11680 | Positive |
| Dia150 | 4689 | Positive | n/a | n/a | 2425 | Positive |
| Dia149 | 10752 | Positive | n/a | n/a | 11004 | Positive |
| Dia148 | 433 | Negative | n/a | n/a | 351 | Negative |
| Dia147 | 68 | Negative | n/a | n/a | 80 | Negative |

n/a: data not available.

**Supplementary Table 7b.** Coefficient of variation for antibody level detection with fingerstick blood following overnight shipment.

| **Sample ID** | **Replicate 1 MFI** | **Replicate 2 MFI** | **Replicate 3 MFI** | **Sample Mean** | **Sample SD** | **CV** |
| --- | --- | --- | --- | --- | --- | --- |
| A44 | 8144 | 8134.5 | 7361 | 7880 | 449 | 5.7% |
| A50 | 9898 | 9576 | 10197 | 9890 | 310 | 3.1% |
| A49 | 7600 | 7542 | 7535 | 7559 | 36 | 0.5% |
| A53 | 15435 | 9696 | 13091 | 12741 | 2886 | 22.7% |
| A54 | 13185 | 12951 | 14418 | 13518 | 788 | 5.8% |
| A91 | 6780 | 7713 | 6779 | 7091 | 539 | 7.6% |
| A93 | 4701 | 4521 | 4023 | 4415 | 351 | 8.0% |
| A81 | 11582 | 13206 | 13661 | 12816 | 1093 | 8.5% |
| A83 | 5252 | 5860 | 5974 | 5695 | 388 | 6.8% |
| A92 | 7853 | 5434 | 6399 | 6562 | 1218 | 18.6% |
|  |  |  | Average Intra-Assay CV | | | 8.7% |

*Fingerstick blood samples shipped overnight and stored for 7 days at room temperature

**Supplementary Table 8a.** IgG positive percent agreement (PPA) for whole blood samples

| Days from Symptom Onset | Number of Samples Tested | QuantiVirus™ Anti-SARS-CoV-2 IgG Test Result | | |
| --- | --- | --- | --- | --- |
|  |  | IgG Positive results | PPA (%) | 95% CI |
| ≥15 days | 31 | 31 | 100.00% | 89.0% to 100.00% |

**Supplementary Table 8b**. IgG negative percent agreement (NPA) for whole blood samples

| Number of Samples Tested | QuantiVirus™ Anti-SARS-CoV-2 IgG Test Result | | |
| --- | --- | --- | --- |
|  | IgG Negative Results | NPA (%) | 95% CI |
| 51 | 51 | 100% | 93.0% to 100.00% |

**Supplementary Table 9**. Microsphere-based anti-SARS-CoV-2 IgG versus PRNT-based neutralizing antibody levels

| Sample ID | Age | Gender | Vaccine vendor | Sample collection after second dose (days) | PRNT50 (NT50) | Fingerstick Blood (MFI) |
| --- | --- | --- | --- | --- | --- | --- |
| Dia021 | 26 | M | Moderna | 22 | 3962 | 11760 |
| Dia022 | 41 | F | Pfizer | 59 | 1705 | 14432 |
| Dia023 | 52 | M | Moderna | 37 | 596 | 8408 |
| Dia024 | 66 | F | Moderna | 84 | 415 | 2944 |
| Dia025 | 55 | F | Moderna | 64 | 7983 | 13286 |
